# Coefficient Inequalities for a Subclass of *p*-Valent Analytic Functions

**DOI:** 10.1155/2014/801751

**Published:** 2014-02-04

**Authors:** Muhammad Arif, Janusz Sokół, Muhammad Ayaz

**Affiliations:** ^1^Department of Mathematics, Abdul Wali Khan University Mardan, Mardan 23200, Pakistan; ^2^Department of Mathematics, Rzeszów University of Technology, Al. Powstancow Warszawy 12, 35-959 Rzeszów, Poland

## Abstract

The aim of this paper is to study the problem of coefficient bounds for a newly defined subclass of *p*-valent analytic functions. Many known results appear as special consequences of our work.

## 1. Introduction

Let *𝒜*(*p*) denote the class of functions *f*(*z*) of the form

(1) $$\begin{array}{c} f\left(z\right)=z^{p}+\sum_{n=p+1}^{\infty }a_{n}z^{n},\;\left(p\in \mathbb{N}\right), \end{array}$$

which are analytic and multivalent in the open unit disk *𝕌* = {*z* : |*z*| < 1}. Also let *𝒮*_p_* and *𝒦*_*p*_ denote the well-known classes of *p*-valent starlike functions and *p*-valent convex functions, respectively.

For *f*(*z*) ∈ *𝒜*(*p*) given by (1) and *g*(*z*) ∈ *𝒜*(*p*) given by

(2) $$\begin{array}{c} g\left(z\right)=z^{p}+\sum_{n=p+1}^{\infty }b_{n}z^{n},\;\left(p\in \mathbb{N}\right), \end{array}$$

the Hadamard product (or convolution) of *f*(*z*) and *g*(*z*) is given by

(3) $$\begin{array}{c} \left(f*g\right)\left(z\right)=z^{p}+\sum_{n=p+1}^{\infty }a_{n}b_{n}z^{n},\;\left(z\in 𝕌\right). \end{array}$$

Motivated by Ruscheweyh operator [1], Goel and Sohi [2] introduced a differential operator *D* ^*δ*+*p*−1^ for *p*-valent analytic functions given by

(4) $$\begin{array}{c} D^{\delta +p-1}f\left(z\right)=\frac{z}{{\left(1-z\right)}^{\delta +p}}*f\left(z\right)=z^{p}+\sum_{n=p+1}^{\infty }\varphi_{n}\left(\delta \right)a_{n}z^{n}, \end{array}$$

with *δ* > −*p*,

(5) $$\begin{array}{c} \varphi_{n}\left(\delta \right)=\frac{{\left(\delta +p\right)}_{n-p}}{\left(n-p\right)!}, \end{array}$$

and (*x*)_*n*_ is a Pochhammer symbol given by

(6) $$\begin{array}{c} {\left(x\right)}_{n}=\{\begin{array}{cc} 1, & n=0,\\ x\left(x+1\right)\left(x+2\right)\cdots \left(x+n-1\right), & n\in \mathbb{N}. \end{array} \end{array}$$

It is obvious that when *δ* is any integer greater than −*p*,

(7) $$\begin{array}{c} D^{\delta +p-1}f\left(z\right)=\frac{z^{p}{\left(z^{\delta -1}f\left(z\right)\right)}^{\left(\delta +p-1\right)}}{\left(\delta +p-1\right)!}. \end{array}$$

The following identity can be easily established:

(8) $$\begin{array}{c} \left(\delta +p\right)D^{\delta +p}f\left(z\right)=\delta D^{\delta +p-1}f\left(z\right)+z{\left(D^{\delta +p-1}f\left(z\right)\right)}^{\prime }. \end{array}$$

Using the generalized Ruscheweyh operator, we define a subclass of *𝒱𝒟*_*p*_^*λ*^(*δ*, *b*, *α*, *β*) of *p*-valent analytic functions as follows.

**Definition 1**

An analytic *p*-valent function *f*(*z*) of the form (1) belongs to the class *𝒱𝒟*_*p*_^*λ*^(*δ*, *b*, *α*, *β*), if and only if

(9) Re{eiλ(1−2b+2bDδ+pf(z)Dδ+p−1f(z))}  >α|2b(Dδ+pf(z)Dδ+p−1f(z)−1)|+βcos⁡λ,

where *α* ≥ 0,  *b* ∈ *ℂ*∖{0}, *δ* > −*p*, *λ* is real with |*λ*| < (*π*/2), and 0 ≤ *β* < 1.

By giving specific values to *α*, *β*, *λ*, *p*, *b*, and *δ* in *𝒱𝒟*_*p*_^*λ*^(*δ*, *b*, *α*, *β*), we obtain many important subclasses studied by various authors in earlier papers; see for details [3–6]; we list some of them as follows:

- *𝒱𝒟*_1_^*λ*^(0,2, 0,0) ≡ *𝒮*_λ_* and *𝒱𝒟*_1_^*λ*^(1,1, 0,0) ≡ *𝒦*_*λ*_, studied by Spacek [7] and Robertson [8], respectively; for the advancement work see [9–11];
- *𝒱𝒟*_1_^0^(0,2, *α*, *β*) ≡ *𝒮𝒟*(*α*, *β*) and *𝒱𝒟*_1_^0^(1,1, *α*, *β*) ≡ *𝒦𝒟*(*α*, *β*), studied by both Owa et al. and Shams et al. [12, 13];
- *𝒱𝒟*_1_^*λ*^(0,2, 1,0) ≡ *𝒰𝒮𝒫*(*λ*) and *𝒱𝒟*_1_^*λ*^(1,1, 1,0) ≡ *𝒰𝒞𝒮𝒫*(*λ*), introduced by Ravichandran et al. [14];
- *𝒱𝒟*_1_^0^(*δ*, *b*, *α*, *β*) ≡ *𝒱𝒟*(*δ*, *b*, *α*, *β*), considered by Latha [15];
- *𝒱𝒟*_1_^0^(0,2, 0, *β*) ≡ 𝒮*(*β*) and *𝒱𝒟*_1_^0^(1,1, 0, *β*) ≡ *𝒦*(*β*), the well-known classes of starlike and convex functions of order *β*.

From the above special cases we note that this class provides a continuous passage from the class of starlike functions to the class of convex functions.

We will assume throughout our discussion, unless otherwise stated, that *α* ≥ 0, 0 ≤ *β* < 1, *δ* > −1, *λ* is real with |*λ*| < (*π*/2), and *b* ∈ *ℂ*∖{0}.

## 2. Main Results

**Theorem 2**

Let *f*(*z*) ∈ *𝒱𝒟*_*p*_^*λ*^(*δ*, *b*, *α*, *β*) with 0 ≤ *α* ≤ *β*. Then *f*(*z*) ∈ *𝒱𝒟*_*p*_^*λ*^(*δ*, *b*, 0, *ζ*), where *ζ* = (*β* − *α*)/(1 − *α*).

**Proof**

Let *f*(*z*) ∈ *𝒱𝒟*_*p*_^*λ*^(*δ*, *b*, *α*, *β*). Then we obtain

(10) Reeiλ{1−2b+2bDδ+pf(z)Dδ+p−1f(z)}  >α|2b(Dδ+pf(z)Dδ+p−1f(z)−1)|+βcos⁡λ  >αReeiλ(1−2b+2bDδ+pf(z)Dδ+p−1f(z))   −αReeiλ+βcos⁡λ,

and this implies

(11) Re[eiλ(1−2b+2bDδ+pf(z)Dδ+p−1f(z))]>ζcos⁡λ.

Also if 0 ≤ *α* ≤ *β*, then we can easily obtain

(12) $$\begin{array}{c} 0\leq \zeta <1, \end{array}$$

and this completes the proof.

**Theorem 3**

If *f*(*z*) ∈ *𝒱𝒟*_*p*_^*λ*^(*δ*, *b*, *α*, *β*), then

(13) $$\begin{array}{c} \left|a_{2}\right|\leq \frac{\left|b\right|\left|\eta \right|}{\left|1-\alpha \right|}, \end{array}$$

and

(14) |an+p−1|≤(δ+p)|b||η|(n−1)|1−α|φn+p−1(δ) ×∏j=1n−2(1+(δ+p)|b||η|j|1−α|), n≥3,

where *φ*_*n*+*p*−1_(*δ*) is given by (5) and

(15) $$\begin{array}{c} \eta =\left(1-\beta \right)\cos\lambda +i\left(1-\alpha \right)\sin\lambda . \end{array}$$

**Proof**

Let *f*(*z*) ∈ *𝒱𝒟*_*p*_^*λ*^(*δ*, *b*, *α*, *β*). Then by Theorem 2, we have

(16) Re[eiλ(1−2b+2bDδ+pf(z)Dδ+p−1f(z))]  >(β−α1−α)cos⁡λ, (z∈E).

Let us define *p*(*z*) by

(17) eiλ(1−2b+2bDδ+pf(z)Dδ+p−1f(z))  =[(1−β1−α)p(z)+(β−α1−α)]cos⁡λ+isinλ.

Then *p*(*z*) is analytic in *E* with *p*(0) = 1 and *Rep*(*z*) > 0, *z* ∈ *E*. Let

(18) $$\begin{array}{c} p\left(z\right)=1+\sum_{n=1}^{\infty }p_{n}z^{n},\;z\in E. \end{array}$$

Then (17) becomes

(19) 1−2b+2bDδ+pf(z)Dδ+p−1f(z)  =1+(1−β)cos⁡λ+i(1−α)sinλeiλ(1−α)∑n=1∞pnzn.

That is,

(20) 2eiλ(1−α)[Dδ+pf(z)−Dδ+p−1f(z)]  =bηDδ+p−1f(z)∑n=1∞pnzn,

where *η* is given by (15). Using (8) in (20), we obtain

(21) 2eiλ(1−α)[z(Dδ+p−1f(z))′−pDδ+p−1f(z)]  =b(δ+p)ηDδ+p−1f(z)∑n=1∞pnzn,

or, equivalently,

(22) 2eiλ(1−α)[∑k=p+1∞(k−p)φk(δ)akzk]  =b(δ+p)η[zp+∑k=p+1∞φk(δ)akzk](∑n=1∞pnzn).

Comparing the coefficients of *z* ^*n*+*p*−1^ on both sides,

(23) 2eiλ(1−α)(n−1)φn+p−1(δ)an+p−1  =b(δ+p)η{p1an+p−2φn+p−2(δ)+⋯+pn−1}.

Taking absolute on both sides and then applying the coefficient estimates |*p*_*n*_| ≤ 2 for Caratheodory functions [3], we have

(24) |an+p−1|≤|b|(δ+p)|η|(n−1)|1−α|φn+p−1(δ) ×{1+φp+1(δ)|ap+1|+⋯+φn+p−2(δ)|an+p−2|}.

We apply mathematical induction on (24). So for *n* = 2,

(25) $$\begin{array}{c} \left|a_{p+1}\right|\leq \frac{\left|b\right|\left|\eta \right|}{\left|1-\alpha \right|}, \end{array}$$

which shows that (13) is true. For *n* = 3,

(26) $$\begin{array}{c} \left|a_{p+2}\right|\leq \frac{\left|b\right|\left|\eta \right|\left(\delta +p\right)}{\left|1-\alpha \right|\left(2\right)\varphi_{p+2}\left(\delta \right)}\left\{1+\varphi_{p+1}\left(\delta \right)\left|a_{p+1}\right|\right\}, \end{array}$$

and using (13), we have

(27) $$\begin{array}{c} \left|a_{p+2}\right|\leq \frac{\left|b\right|\left|\eta \right|\left(\delta +p\right)}{\left(1-\alpha \right)\left(2\right)\varphi_{p+2}\left(\delta \right)}\left\{1+\frac{\left|b\right|\left|\eta \right|\left(\delta +p\right)}{\left(1-\alpha \right)}\right\}. \end{array}$$

Therefore, (14) holds for *n* = 3.

Assume that (14) is true for *n* = *k*; that is,

(28) |ak+p−1|  ≤|b||η|(δ+p)|1−α|(k−1)φk+p−1(δ)∏j=1k−2(1+|b||η|(δ+p)|1−α|j).

Consider

(29) |ak+p|≤|b||η|(δ+p)|1−α|(k)φk+p(δ) ×{(1+|b||η|(δ+p)|1−α|)+|b||η|(δ+p)|1−α|(2)   ×(1+|b||η|(δ+p)|1−α|)   +⋯+|b||η|(δ+p)|1−α|(k−1)   ×∏j=1k−2(1+|b||η|(δ+p)|1−α|j)}=|b||η|(δ+p)|1−α|(k)φk+p(δ)∏j=1k−1(1+|b||η|(δ+p)|1−α|j).

Therefore, the result is true for *n* = *k* + 1, and hence by using mathematical induction, (14) holds true for all *n* ≥ 3.

If we put *λ* = 0, *p* = 1, *b* = 2, and *δ* = 0 in Theorem 3, we obtain the result proved in [12].

**Corollary 4**

If *f*(*z*) ∈ *𝒮𝒟*(*α*, *β*), then

(30) $$\begin{array}{c} \left|a_{2}\right|\leq \frac{2\left(1-\beta \right)}{\left|1-\alpha \right|},\\ \left|a_{n}\right|\leq \frac{2\left(1-\beta \right)}{\left(n-1\right)\left|1-\alpha \right|}\prod_{j=1}^{n-2}\left(1+\frac{2\left(1-\beta \right)}{j\left|1-\alpha \right|}\right),\;\left(n\geq 3\right). \end{array}$$

If one takes *α* = 0 in Corollary 4, one obtains the following inequality:

(31) $$\begin{array}{c} \left|a_{n}\right|\leq \frac{1}{\left(n-1\right)!}\prod_{j=2}^{n}\left(j-2\beta \right),\;\left(n\geq 2\right), \end{array}$$

which was proved by Robertson [16].

By setting *λ* = 0, *p* = 1, *b* = 1, and *δ* = 1 in Theorem 3, one obtains the result proved in [12].

**Corollary 5**

If *f*(*z*) ∈ *𝒦𝒟*(*α*, *β*), then

(32) $$\begin{array}{c} \left|a_{2}\right|\leq \frac{\left(1-\beta \right)}{\left|1-\alpha \right|},\\ \left|a_{n}\right|\leq \frac{2\left(1-\beta \right)}{n\left(n-1\right)\left|1-\alpha \right|}\prod_{j=1}^{n-2}\left(1+\frac{2\left(1-\beta \right)}{j\left|1-\alpha \right|}\right),\;\left(n\geq 3\right). \end{array}$$

Letting *α* = 0 in Corollary 5, one gets the following inequality proved by Robertson [16]:

(33) $$\begin{array}{c} \left|a_{n}\right|\leq \frac{1}{n!}\prod_{j=2}^{n}\left(j-2\beta \right),\;\left(n\geq 2\right). \end{array}$$

**Theorem 6**

If *f*(*z*) ∈ *𝒜*(*p*) and satisfies

(34) 2(α+1)(δ+p)|b|sinλ2+β(δ+p)|b|cos⁡λ   +∑k=p+1∞φk(δ)|ak|   ×[(α+1){2(δ+p)|b|sinλ2+2(k−p)}     −β(δ+p)|b|cos⁡λ+(δ+p)|b|]  <(δ+p)|b|,

where *φ*_*k*_(*δ*) is given by (5), then *f*(*z*) ∈ *𝒱𝒟*_*p*_^*λ*^(*δ*, *b*, *α*, *β*).

**Proof**

Suppose (34) holds. Also let us suppose

(35) $$\begin{array}{c} Q\left(z\right)=e^{i\lambda }\left(1-\frac{2}{b}+\frac{2}{b}\frac{D^{\delta +p}f\left(z\right)}{D^{\delta +p-1}f\left(z\right)}\right). \end{array}$$

Then

(36) |Q(z)−1| =|(beiλ−2eiλ−b)Dδ+p−1f(z)+2eiλDδ+pf(z)bDδ+p−1f(z)|.

Using (8) and then simplifications gives

(37) |Q(z)−1| =|({bδ(eiλ−1)+bp(eiλ−1)−2peiλ}   ×Dδ+p−1f(z)+2eiλz(Dδ+p−1f(z))′)   ×(b(δ+p)Dδ+p−1f(z))−1| ≤(2(δ+p)|b|sinλ2+|∑k=p+1∞φk(δ)akzk|    ×[2(δ+p)|b|sinλ2+(k−p)])   ×((δ+p)|b|(1−∑k=p+1∞φk(δ)|ak|))−1.

Now consider

(38) α|Q(z)−1|−Re|Q(z)−1|+βcos⁡λ ≤(α+1)|Q(z)−1|+βcos⁡λ ≤(((α+1){2(δ+p)|b|sinλ2+|∑k=p+1∞φk(δ)akzk|        ×[2(δ+p)|b|sinλ2+(k−p)]})   ×((δ+p)|b|(1−∑k=p+1∞φk(δ)|ak|))−1)  +βcos⁡λ.

The last expression is bounded by 1 if

(39) 2(α+1)(δ+p)|b|sinλ2+β(δ+p)|b|cos⁡λ  +∑k=p+1∞φk(δ)|ak|   ×[(α+1){2(δ+p)|b|sinλ2+2(k−p)}    −β(δ+p)|b|cos⁡λ+(δ+p)|b|]<(δ+p)|b|,

and this completes the proof.

For *λ* = 0, *δ* = −*p* + 1, *b* = 2, and *α* = 0 in Theorem 6, we obtain the following.

**Corollary 7**

If *f*(*z*) ∈ *𝒜*(*p*) and satisfies

(40) $$\begin{array}{c} \sum_{k=p+1}^{\infty }\left(k+1-p-\beta \right)\left|a_{k}\right|<\left(1-\beta \right), \end{array}$$

then *f*(*z*) ∈ *𝒮*_p_*(*β*), the class of *p*-valent starlike functions of order *β*.

For *λ* = 0, *δ* = −*p* + 2, *b* = 1, and *α* = 0 in Theorem 6, one has the following.

**Corollary 8**

If *f*(*z*) ∈ *𝒜*(*p*) and satisfies

(41) $$\begin{array}{c} \sum_{k=p+1}^{\infty }\left(k-p+1\right)\left(k+1-p-\beta \right)\left|a_{k}\right|<\left(1-\beta \right), \end{array}$$

then *f*(*z*) ∈ *𝒦*_*p*_(*β*), the class of *p*-valent convex functions of order *β*.

Further for *p* = 1 in both the last two corollaries, one obtains the results for the classes 𝒮*(*β*) and *𝒦*(*β*) which was proved by Merkes et al. [17] and Silverman [18], respectively.
